# Cardiorespiratory and muscle oxygenation responses to voluntary hypoventilation at low lung volume in upper body repeated sprints

**DOI:** 10.1007/s00421-024-05569-1

**Published:** 2024-08-13

**Authors:** Cristóvão H. Rosa, Cristina P. Monteiro, Cláudia Barata, Mário C. Espada, Maria João Valamatos, André Bento, Ricardo J. Minhalma, Joana Filipa Reis

**Affiliations:** 1 Laboratory of Physiology and Biochemistry of Exercise, Faculdade de Motricidade Humana, Universidade de Lisboa, Oeiras, Portugal; 2 CIPER, Faculdade de Motricidade Humana, Universidade de Lisboa, Lisbon, Portugal; 3 Instituto Politécnico de Setúbal, Escola Superior de Educação, Setúbal, Portugal; 4 Neuromuscular Research Laboratory, Faculdade Motricidade Humana, Universidade Lisboa, Oeiras, Portugal; 5 Sport Physical Activity and Health Research & Innovation Center (SPRINT), Rio Maior, Portugal; 6 Comprehensive Health Research Centre (CHRC), University of Évora, Évora, Portugal; 7 Life Quality Research Centre (CIEQV), Setúbal, Portugal; 8 Escola Superior de Educacao e Comunicacao, Universidade do Algarve, Faro, Portugal; 9 Portugal Football School, Portuguese Football Federation, FPF, Cruz-Quebrada, Portugal

**Keywords:** Arm cycle ergometer, Voluntary hypoventilation with low lung volume, End-expiratory breath-hold, Hypoxia, Repeated sprinting

## Abstract

**Purpose:**

To investigate the impact of voluntary hypoventilation at low lung volumes (VHL) during upper body repeated sprints (RS) on performance, metabolic markers and muscle oxygenation in Brazilian Jiu-Jitsu (BJJ) athletes.

**Methods:**

Eighteen male well-trained athletes performed two randomized RS sessions, one with normal breathing (RSN) and another with VHL (RS-VHL), on an arm cycle ergometer, consisting of two sets of eight all-out 6-s sprints performed every 30 s. Peak (PPO), mean power output (MPO), and RS percentage decrement score were calculated. Arterial oxygen saturation (SpO_2_), heart rate (HR), gas exchange, and muscle oxygenation of the long head of the triceps brachii were continuously recorded. Blood lactate concentration ([La]) was measured at the end of each set. Bench press throw peak power (BP_PP_) was recorded before and after the RS protocol.

**Results:**

Although SpO_2_ was not different between conditions, PPO and MPO were significantly lower in RS-VHL. ${\dot{\text{V}}}$_E_, HR, [La], and RER were lower in RS-VHL, and VO_2_ was higher in RS-VLH than in RSN. Muscle oxygenation was not different between conditions nor was its pattern of change across the RS protocol influenced by condition. [La] was lower in RS-VHL than in RSN after both sets.

**Conclusion:**

Performance was significantly lower in RS-VHL, even though SPO_2_ was not consistent with hypoxemia. However, the fatigue index was not significantly affected by VHL, nor was the neuromuscular upper body power after the RS-VHL protocol. Additionally, [La] was lower, and oxygen consumption was higher in RS-VHL, suggesting a higher aerobic contribution in this condition.

## Introduction

Voluntary hypoventilation at low lung volumes (VHL) consists of an end-expiratory breath-hold at functional residual capacity (Woorons 2014). Accordingly, subjects can only sustain it for a brief period, imposing an intermittent pattern to its application where breath holds alternate with recovery periods of normal breathing (Woorons 2014). When combined with large muscle mass exercise, VHL induces severe arterial oxygen desaturation, leading to muscular and cerebral deoxygenation (Kume et al. 2016; Woorons et al. 2007, 2010, 2011), causing both hypoxia and hypercapnia (Woorons 2014; Woorons et al. 2010). In this regard, VHL is considered an intermittent hypoxic method within the “Living Low-Train High” hypoxic model (Girard et al. 2020; Millet et al. 2019). During moderate exercise, when compared with unimpaired ventilation, VHL induced higher blood lactate concentration ([La]) (Kume et al. 2016) and lower muscle oxygenation (Kume et al. 2016), suggesting a greater energy supply from the anaerobic glycolysis. Nonetheless, the effects of combining VHL with repeated-sprint (RS) exercise have only recently been studied, with authors reporting greater oxygen uptake (${\dot{\text{V}}}$O_2_) (Woorons et al. 2017, 2019a) and lower [La] at the end of the exercise, which the authors attributed to better lactate clearance (Woorons et al. 2017, 2019b). Given that the glycolytic pathway may be improved with RS-VHL, activities that predominantly use this energy pathway are more likely to benefit from this method.

Brazilian Jiu-Jitsu (BJJ) is included in this category. This activity is a grappling combat sport with intermittent energetic demands, characterized by short bouts of high-intensity efforts and explosive movements interspersed with periods of moderate or low-intensity activity (Andreato et al. 2017). Although predominantly aerobic, post-match samples of [La] (≈10 mmol L^−1^) suggest a moderate-to-high glycolytic activation (Andreato et al. 2016).

All the previous studies that have used repeated-sprint exercise in combination with VHL (RS-VHL) were performed in running (Fornasier-Santos et al. 2018; Lapointe et al. 2020), cycling (Woorons et al. 2019b, 2020), or swimming (Woorons et al. 2016; Trincat et al. 2017), all activities using a large muscle mass. In combat sports, more specifically those who use *gi* (e.g., BJJ and Judo), it was reported that the upper limbs are more likely to have local fatigue between matches and throughout the competition when compared with lower limbs (Bonitch-Góngora et al. 2012; Andreato et al. 2017; Franchini et al. 2018a, b; Kons et al. 2018). Furthermore, the upper body resistance to fatigue is an indicator that distinguishes experienced from novice athletes or medallists from non-medallists in international events (Andreato et al. 2017; Kons et al. 2018).

A recent study by Willis et al. (2019a, b) found that arms were more sensitive to local deoxygenation compared to legs during an RS protocol in an arm ergometer, with induced systemic hypoxia or with blood flow restriction. Beard et al. (2019) found improvements in repeated-sprint ability after an upper limb repeated sprint in hypoxia (RSH) training period, when compared with the same training protocol in normoxia. Such results suggest a potential use of the VHL technique combined with upper limb exercise, although research focused on VHL is notably scarce, particularly in combat sports. Furthermore, the impact of such training sessions on neuromuscular performance has yet to be established and could provide important information on integrating this technique with specific training sessions of power sports.

Hence, this study aimed to compare the acute effects of an upper body RS-VHL protocol with the same exercise protocol without breathing impairment (RSN) in BJJ fighters. Additionally, we propose to analyse the impact of the RS-VHL protocol on explosive movements, comparing bench press peak power (BP_PP_) before and after the RS protocol in both conditions.

## Methods

### Participants

Eighteen well-trained male BJJ athletes (aged 32 ± 7.3 years; body mass 73.8 ± 10.8 kg; 174.9 ± 5.5 cm) with 8.9 ± 4.2 years of competition at international and/or national events completed this study.

The study was approved by the Ethics Committee of the Faculdade de Motricidade Humana, Universidade de Lisboa (CEIFMH—nr. 42/2021). The volunteers signed an informed consent, filled out a health questionnaire (Physical Activity Readiness Questionnaire, PAR-Q & YOU), and had their arterial blood pressure measured (Hartmann Veroval^®^, Heidenheim, Germany). Exclusion criteria included: (1) report of any cardiorespiratory or cardiovascular known disease; (2) any affirmative answer from the written PAR-Q & YOU; or (3) a systolic pressure above 140 mm/Hg and a diastolic pressure above 90 mm/Hg (American College of Sports Medicine 2018).

### Experimental design

**Fig. 1:**
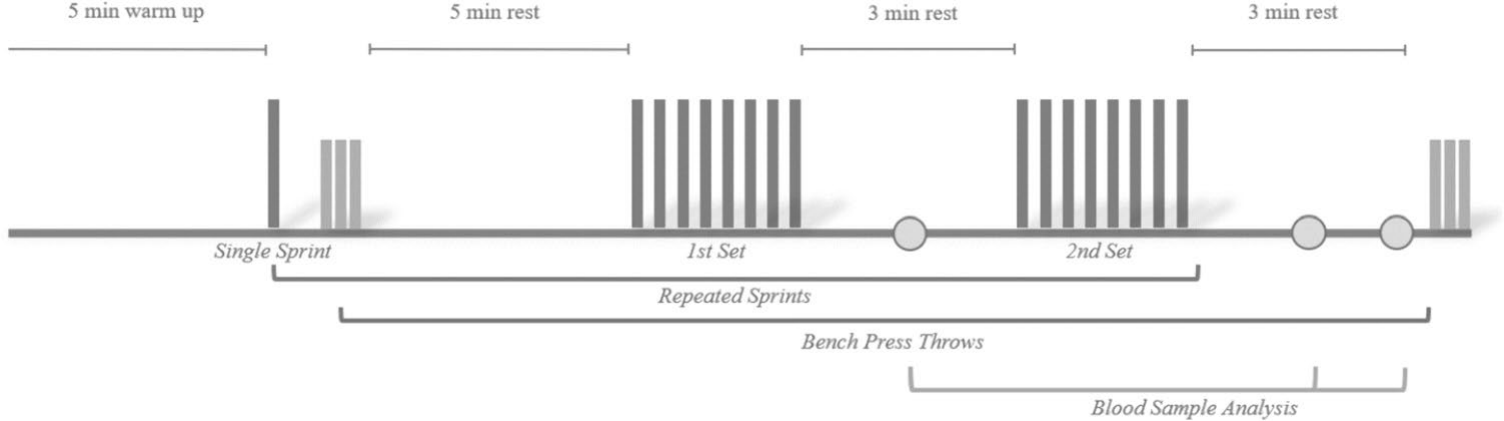
Experimental design overview of the repeated sprints sessions

This was an experimental study with a randomized crossover design. Each athlete participated in three exercise sessions: a preliminary session where participants performed a maximal graded exercise test to assess peak oxygen consumption (${\dot{\text{V}}}$O_2peak_) and familiarized themselves with the testing procedures and the VHL technique, and two other exercise sessions for RS evaluation, one using RSN and the other using RS-VHL. The RS sessions were randomly assigned and separated by at least 48 h within 15 days (Woorons et al. 2017). Participants were asked to avoid vigorous exercise, alcohol drinking, and caffeine 24 h before every test session and to maintain their regular diet throughout the study. All test sessions were performed approximately at the same time of day (±2 h) to avoid any potential effects of chronobiological variability on physiological responses (Pullinger et al. 2020; Fig. 1).

In the RS evaluation sessions, performance indicators, pulmonary gas exchange, heart rate (HR), arterial oxygen saturation (SpO_2_), and muscle oxygenation of the triceps brachii were evaluated during the RS protocols, and neuromuscular fatigue was assessed by measuring BP_PP_ before and after the RS protocols. Rating of perceived exertion (RPE) and [La] were recorded during and at the end of the RS protocols. All the tests were performed on an arm-crank ergometer (Lode Angio, Groningen, The Netherlands). The configuration for each participant was replicated in every session. Participants remained seated with their shoulder joint aligned with the pedal crank axle.

#### Familiarization with the VHL technique

To familiarize the athletes with the VHL technique while arm-pedaling with no additional load (0 W), participants were asked to perform an end-expiratory breath-hold and to keep it until the first sign or urge to breathe; this exercise was then interspersed by long and deep unforced breaths. This was performed while controlling each inhalation and exhalation, for about 5 min. For the next 5 min, participants were asked to do the same procedure but to go further toward their limit while keeping the end-expiratory breath-hold and accelerating the pedaling rhythm during the breath holds. After these exercises, all subjects participated in four 6-s all-out sprints with 24-s passive rest while using VHL technique, simulating the experimental protocol.

#### Peak oxygen uptake

To assess the ${\dot{\text{V}}}$O_2peak_, participants performed a maximal graded exercise test using an arm-crank ergometer. The protocol began with a 3-min warm-up with no load (0 W). After that, a load of 15 W was applied and increased by 15 W increments every minute until volitional exhaustion was attained (Antunes et al. 2022). Throughout the test, the participants were required to maintain a cadence of 70 ± 5 rpm (Price et al. 2007). All participants were given strong verbal encouragement during the test and were asked to remain seated to diminish any compensatory torso movements.

During the whole protocol, pulmonary gas exchange (${\dot{\text{V}}}$O_2_, carbon dioxide, and respiratory exchange ratio, ${\dot{\text{V}}}$CO_2_, RER, respectively), ventilation (${\dot{\text{V}}}$_E_), and HR data were continuously monitored. The ${\dot{\text{V}}}$O_2peak_ was calculated as the highest 30-s ${\dot{\text{V}}}$O_2_ average attained during the protocol (Antunes et al. 2022).

#### Repeated-sprint protocol

Before starting the protocol, subjects performed a 5-min warm-up at low-intensity pedaling in the arm crank with no additional load while maintaining a cadence of 70 ± 5 rpm. Afterward, torque was set as the result of body weight times the distance between the midpoint of the pedal and the crank axle, as specified by the manufacturer, and a single 6-s all-out sprint was performed. The peak power output (PPO) was recorded to serve as a reference for the first sprint of the RS test protocol.

Testing began after a 5-min period of rest. The RS test protocol consisted of two sets (set 1 and set 2) of eight “all-out” 6-s sprints on the arm cycle ergometer. The 6-s sprints were separated by 24-s of passive recovery and 3 min of passive rest between the two sets (Woorons et al. 2017). In the first sprint of set 1, subjects were expected to achieve at least 90% of the PPO reached in the single sprint performed prior to the 5-min resting period. If this was not achieved, they restarted the set after a new 5-min period of rest (Woorons et al. 2017; Willis et al. 2019a, b).

Participants were strongly encouraged verbally and instructed to remain seated during RS protocol. To ensure the correct application of the VHL technique throughout the entire protocol, all participants were notified 5 s before the start of each sprint, and 2 s before the start, they were given specific instructions to exhale and hold their breath. Throughout both RS protocols, PPO, mean power output (MPO) and total work (TW), pulmonary gas exchange (${\dot{\text{V}}}$O_2_, ${\dot{\text{V}}}$CO_2_, RER), ${\dot{\text{V}}}$_E_, HR, SpO_2_, and muscle oxygenation (oxyhemoglobin, deoxyhemoglobin, and tissue oxygenation index, respectively, O_2_Hb, HHb, and TOI) of the triceps brachii data were continuously monitored.

### Measurements

#### Participants’ characterization

During the familiarization session, participants body mass (Seca, Model 761, Hamburg, Germany) and stature were measured (Harpenden, Holtain Ltd, Crosswell, UK) to the nearest 1.0 kg and 0.1 cm, respectively. Handgrip isometric strength (HG_Iso_) of the dominant hand was also measured twice, separated by 1 min rest. Participants were asked to stand with the dominant arm extended along the trunk and to squeeze the dynamometer (Jamar, Lafayette, CA, USA) with the greatest possible force for 3–5 s. The highest value of the two repetitions was recorded.

#### Performance

The PPO and MPO were calculated for each of the 6-s sprints and TW was calculated as the result of the averaged power obtained in each of the 6-s sprints times the duration of the sprint (6-s).

Fatigue in each set was evaluated by calculating repeated-sprint percentage decrement score (RSA_decs_) as follows:

$${\text{RSAdecs}} = (100 \times ({\text{Sum}}\,{\text{of}}\,{\text{MPO}}/{\text{Ideal}}\,{\text{MPO}}\,{\text{of}}\,{\text{the}}\,{\text{set}}))--100,$$

where Sum of MPO = sum of MPO from all sprints of the set, and Ideal MPO of the set = highest MPO from all the sprints of the set × number of sprints of the set (Woorons et al. 2017).

#### Arterial oxygen saturation

The SpO_2_ was continuously measured during the RS protocols using a pulse oximeter placed on the ear lobe (Nonin PureSAT^®^ SpO_2_ technology, WristOx2 3150 USB, Plymouth, USA), which was taped to diminish possible light interference and to ensure that the sensor was fixed throughout the protocol. Data were obtained once per second and averaged and analysed over 6 s. To account for the delay observed in the drop of the values, the lowest value obtained during or just after each sprint was used for further analysis (Woorons et al. 2017).

#### Gas exchange and heart rate

Throughout the whole RS protocols, ${\dot{\text{V}}}$_E_, ${\dot{\text{V}}}$O_2_, and ${\dot{\text{V}}}$CO_2_ were collected breath-by-breath with a gas analyzer (MetaMax 3Br2, Cortex Biophysik, Leipzig, Germany) after calibration according to the manufacturer’s instructions. The HR was continuously recorded and monitored using an HR sensor (Polar^®^ H10, Kempele, Finland). To account for the absence of data during breath holdings in the RS-VHL protocol, for both protocols, ${\dot{\text{V}}}$_E_, ${\dot{\text{V}}}$O_2_, ${\dot{\text{V}}}$CO_2_, RER, and HR data were analysed from the end of each sprint until the beginning of the next sprint and in the 24 s after the end of the last sprint of each set. Averages over 6 s were calculated, and the highest values of ${\dot{\text{V}}}$O_2_ (mL·kg^−1^·min^−1^), ${\dot{\text{V}}}$CO_2_ (L·min^−1^), ${\dot{\text{V}}}$_E_ (L·min^−1^), RER, and HR (bpm) were used for analysis.

#### Muscle oxygenation

Throughout the RS protocols, changes in O_2_Hb and HHb concentrations and TOI from baseline were measured by near-infrared spectroscopy (NIRS) (Niro 200, Hamamatsu, Japan). The skin was shaved and the NIRS probe was placed on the belly of the long head of the triceps brachii, because this muscle participates in both elbow and shoulder extension; an elastic non-compressive bandage was wrapped around the probe and arm to prevent movement of the probe and any potential source of light interference. A pen and photographic record were used to mark the probe placement for test replication. Basal tissue oxygenation values were obtained after 1 min at rest while sitting on the chair before warm-up in each condition. To attain for any possible delay in the response of muscle oxygenation variables, data were averaged over 6 s and analysed from the beginning of each sprint to the beginning of the next or to the 24th second after the end of the last sprint, in each set. The lowest values of O_2_Hb and TOI and the highest values of HHb in each sprint were kept for analysis.

#### Rating of perceived exertion and blood lactate concentration

In both exercise conditions, RPE was obtained immediately after the end of each set using the Modified Borg Scale (range 0–10) (Woorons et al. 2017).

[La] were obtained using a Lactate Pro device (Arkray, Kyoto, Japan). A blood sample from the ear lobe was collected by capillary puncture 1.5 min after the end of both set 1 and set 2 and 3 min after the end of set 2. The highest value of the two samples collected at the end of set 2 was kept for analysis (Woorons et al. 2017).

#### Neuromuscular fatigue

In each RS session, before and after the RS protocol, the participants’ BP_PP_ was measured to assess neuromuscular fatigue. Before the RS protocols, two sets of three throws were executed 1 min and 30 s apart, the first set (warm-up) with 25% and the second (main set) with 50% of the body weight. Only the main set was performed after the RS protocols. Chronojump Boscosystems^®^ linear encoder and Chronojump software version 1.9.0 (Chronojump, Barcelona, Spain) were used to measure and record BP_PP_ of each repetition, and the best repetitions of the main sets were kept for analysis.

### Statistical analysis

Statistical analysis was performed using IBM SPSS Statistics (Version 28.0, IBM, NY) and R software (Version 4.2.0, Open-Source Code, General Public License). Based on an effect size of 0.4 and power of 0.8, a priori power analysis (GPower Version 3.1.9.3) for two-way repeated-measures analysis of variance suggested a total of 14 participants. All the results are expressed as mean ± SD. The data were analysed using a general linear model for repeated-measures analysis of variance (ANOVA), considering the condition of O_2_ availability (RS-VHL or RSN), sets (set 1 and set 2), and sprints (from 1 to 8) as three within factors. Huynh–Felt correction was used to attain eventual violations of sphericity in the sprint factor. For RSA_decs_, [La], and RPE, analysis of variance (ANOVA) was performed using a general linear model for repeated measures considering the condition of O_2_ availability (RS-VHL or RSN) and sets (set 1 and set 2) as two within factor. When an interaction between the within factors was observed, pairwise comparisons with Bonferroni adjustment were performed to assess simple main effects. If normal distribution was not verified, the nparLD module of the R software was used to perform a non-parametric two-way ANOVA-type test, and Wilcoxon signed-ranks tests were used to assess simple main effects and differences between conditions for complete protocols. Significance (*p*) was set at 0.05.

## Results

The ${\dot{\text{V}}}$O_2peak_ of the participants in the maximal arm-crank graded test was 34.1 ± 7.44 ml kg^−1^ min^−1^, and their HG_Iso_ was 51.0 ± 11.6 kg.

### Performance

**Fig. 2:**
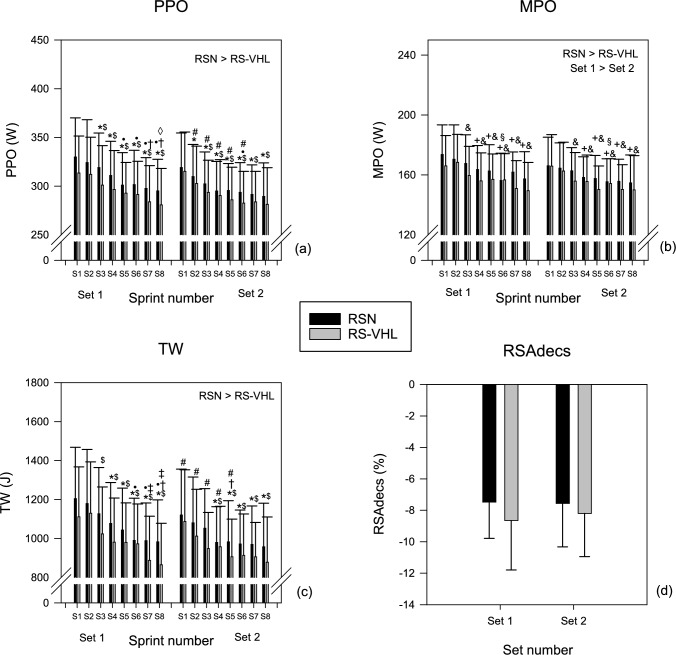
Peak power output (PPO) (**a**) and mean power output (MPO) (**b**) achieved and total work produced (TW) (**c**) at each sprint of the two sets, and repeated-sprint percentage decrement score (RSA_decs_) (**d**) at the end of each set of the repeated-sprint exercise performed with voluntary hypoventilation at low lung volume (RS-VHL) and with normal breathing (RSN). * Significantly different from sprint 1 in the same set; ^$^ significantly different from sprint 2 in the same set; ^•^ significantly different from sprint 3 in the same set; ^†^ significantly different from sprint 4 in the same set; ^‡^ significantly different from sprint 5 in the same set; ^◊^ significantly different from sprint 5 in the same set; ^+^ significantly different from S1 independently of set; & significantly different from S2 independently of set; ^§^ significantly different from S2 independently of set; ^#^ significantly different from the sprint with the same number in set 1

Performance results are presented in Fig. 2.

Independently of set or sprint, PPO, MPO, and TW values were significantly lower in RS-VHL compared to RSN (PPO: RS-VHL—294.3 ± 8.40 W, RSN—304.8 ± 7.83 W, *F*(1, 16) = 18.740, *p* < 0.001; MPO: RS-VHL—156.8 ± 4.08 W, RSN—161.8 ± 3.66 W, *F*(1, 16) = 12.752, *p* = 0.003; TW: RS-VHL—972.6 ± 49.59 W, RSN—1044 ± 49.06 W, *F*(1, 16) = 25.586, *p* < 0.001). Although there was no significant main effect of condition, there was a gradual decline in performance indicators from the 1st sprint of each set with different profiles between sets for PPO (Fig. 2a) and TW (Fig. 2b). In detail, for PPO, in the 1st set, the value of the 1st sprint was higher than that of the 3rd sprint and subsequent sprints, while in the 2nd set, the value of the 1st sprint was higher than that of all subsequent sprints; for TW, although the value of the 1st sprint was higher than that of the 4th sprint and subsequent sprints in both sets, the value of the 2nd sprint was higher than that of the 3rd sprint and subsequent sprints in the 1st set, while it was higher than that of the 4th sprint and subsequent sprints in the 2nd set. The drop of both indicators from the 3rd sprint to the last also differed between sets. For MPO (Fig. 2c), in the 1st sprint, the value was higher than that in the 4th sprint and subsequent sprints, the value in the 2nd sprint was higher than that in all the subsequent sprints, and the value in the 3rd sprint was higher than in the 6th sprint. Comparing sets in each sprint shows that PPO values in sprints 2–6 and TW values in sprints 1–5 were lower in the 2nd than in the 1st set. MPO values in the 2nd set (157.5 ± 3.71 W) were lower than in the 1st set (161.1 ± 3.98 W) independently of the sprint (*F*(1, 16) = 14.346, *p* = 0.002).

For RSA_decs_, there was no significant interaction between sets and conditions nor significant main effects of sets or conditions (Fig. 2d).

### Arterial oxygen saturation

**Fig. 3:**
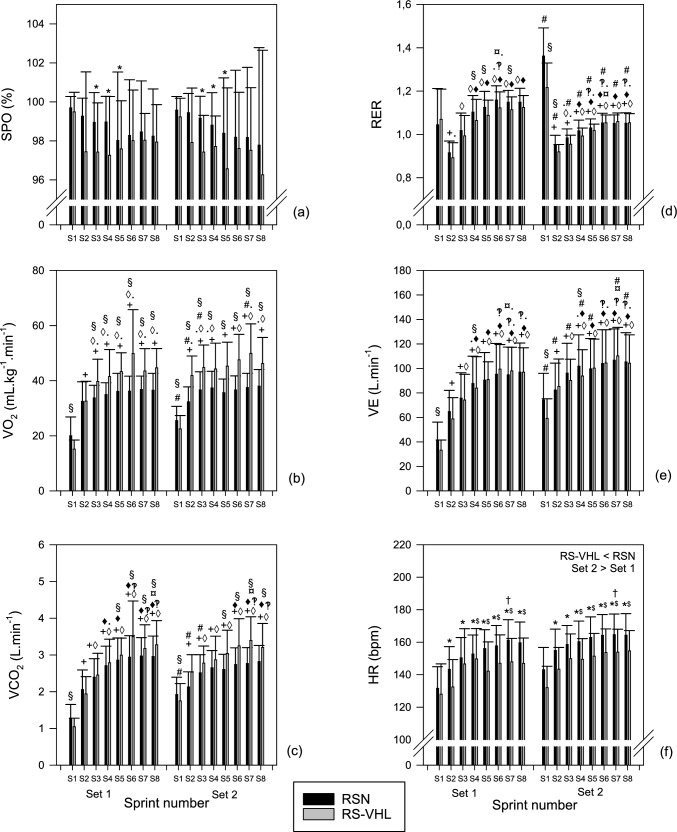
Minimum arterial oxygenation (SpO_2_) (**a**) measured during or just after each sprint of the two sets of the repeated-sprint exercise performed with voluntary hypoventilation at low lung volume (RS-VHL) and with normal breathing (RSN). Maximum values of oxygen uptake (VO_2_) (**b**), carbon dioxide production (VCO_2_) (**c**), respiratory exchange ratio (RER) (**d**), ventilation (VE) (**e**), and heart rate (HR) (**f**) measured over the recovery period of each sprint during the RS-VHL and RSN protocols. * Significantly different from sprint 1 independently of condition or set; ^$^ significantly different from sprint 2 independently of condition or set; ^†^ significantly different from sprint 4 independently of condition or set; ^#^ significantly different from the sprint with the same number in set 1 independently of condition; ^#^ significantly different from the sprint with the same number in set 1 independently of condition; ^+^ Significantly different from sprint 1 in the same set, both in RSN and RS-VHL; ^◊^ significantly different from sprint 2 in the same set, both in RSN and RS-VHL; ^◊^ significantly different from sprint 2 in the same set in RSN; ^◊^ significantly different from sprint 2 in the same set in RS-VHL; ^♦^ significantly different from sprint 3 in the same set, both in RSN and RS-VHL; ^♦^ significantly different from sprint 3 in the same set in RSN; ^♦^ significantly different from sprint 3 in the same set in RS-VHL; ^‽^ significantly different from sprint 4 in the same set, both in RSN and RS-VHL; ^‽^ significantly different from sprint 4 in the same set in RS-VHL; ^¤^ significantly different from sprint 5 in the same set, both in RSN and RS-VHL; ^¤^ significantly different from sprint 5 in the same set in RS-VHL

Independently of set or condition, the minimum values of SpO_2_ attained in the 3rd, 4th, and 5th sprint were lower than the value of the 1st sprint (Fig. 3a). There were no significant differences between conditions in SpO_2_.

### Gas exchange and heart rate

Pulmonary gas exchange variables and HR results are presented in Fig. 3.

In both sets, the maximum ${\dot{\text{V}}}$O_2_ after the 1st sprint was lower in RS-VHL than in RSN. Additionally, from the 3rd sprint onwards in the 1st set and from the 2nd sprint onwards in the 2nd set, the values were higher in RS-VLH than in RSN. Afterwards, VO_2_ gradually increased in both conditions till sprint 6 or 7, depending on the set or condition (see Fig. 3b).

In both conditions, after the 1st and the 3rd sprints, as well as in RS-VHL after the 2nd and the 7th sprints, maximum ${\dot{\text{V}}}$O_2_ values in the 1st set were lower than those in the 2nd set.

Independently of set or condition, maximum ${\dot{\text{V}}}$CO_2_ after the sprints increased progressively but with different patterns (Fig. 3c). As a consequence, maximum ${\dot{\text{V}}}$CO_2_ values after the 1st sprint were lower in RS-VHL than in RSN. Still, after the 5th and subsequent sprints, they were higher in RS-VHL than in RSN. Regarding differences between sets, sprints 1–3 in the 1st set were lower than in the 2nd set.

The maximum values of RER after the sprints decreased from the 1st to the 2nd sprints and then gradually increased with different patterns depending on set or condition (Fig. 3d). As a result, they were lower in RS-VHL in the second half of the 1st set (sprints 4, 5, and 7) and in the beginning of the 2nd set (sprints 1 and 2), and, in both conditions at the start of the sets, they were lower in the 1st set than in the 2nd.

Maximum values of ${\dot{\text{V}}}$_E_ increased gradually until sprint 4 in RSN and until sprint 7 in RS-VHL, and the values of each sprint in the 1st set were lower than the values of the corresponding sprint in the 2nd set (Fig. 3e).

Independently of condition or set, HR increased gradually until sprint 4 (Fig. 3f). HR was lower in RS-VHL (145.6 ± 3.00 bpm) than in RSN (155.4 ± 2.81 bpm), (*F*(1,16) = 20.240, *p* < 0.001) and higher in set 2 (153.9 ± 2.71 bpm) than in set 1 (147.1 ± 2.81 bpm) (*F*(1,16) = 33.894, *p* < 0.001).

### Muscle oxygenation

**Fig. 4:**
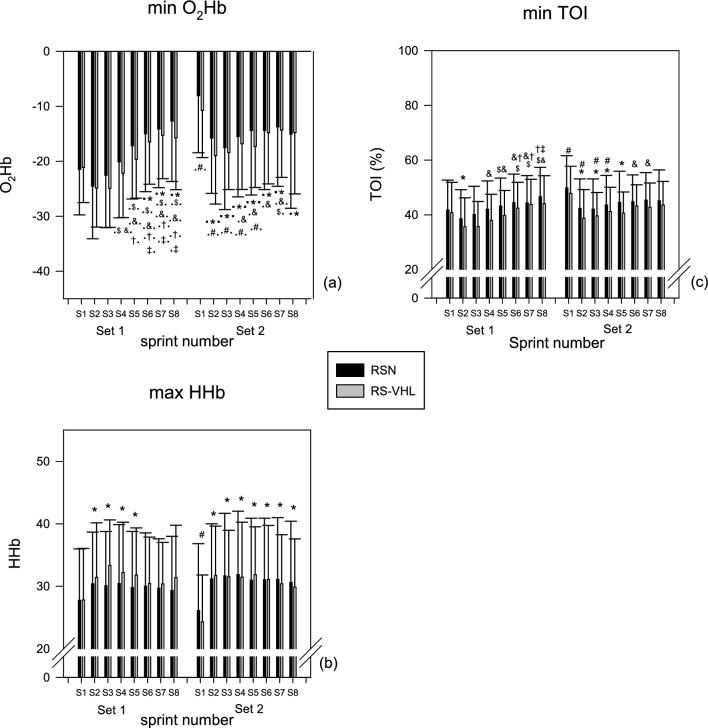
Minimum value of change of muscle oxygenated haemoglobin/myoglobin (O_2_Hb) (**a**) and tissue oxygenation index (TOI) (**b**), and maximum value of change of muscle deoxygenated haemoglobin/myoglobin (HHb) (**c**) observed during or just after each sprint of the two sets of the repeated-sprint exercise performed with voluntary hypoventilation at low lung volume (RS-VHL) and with normal breathing (RSN). * Significantly different from sprint 1 in the same set, independently of condition; ^$^ significantly different from sprint 2 in the same set, independently of condition; ^&^ significantly different from sprint 3 in the same set, independently of condition; ^†^ significantly different from sprint 4 in the same set, independently of condition; ^‡^ significantly different from sprint 5 in the same set independently of condition; ^#^ significantly different from the sprint with the same number in set 1 independently of condition; * significantly different from sprint 1 in the same set, both in RSN and RS-VHL; * significantly different from sprint 1 in the same set in RSN; ^$^ significantly different from sprint 2 in the same set, both in RSN and RS-VHL; ^$^ significantly different from sprint 2 in the same set in RSN; ^$^ significantly different from sprint 2 in the same set in RS-VHL; ^&^ significantly different from sprint 3 in the same set, both in RSN and RS-VHL; ^&^ significantly different from sprint 3 in the same set in RSN; ^&^ significantly different from sprint 3 in the same set in RS-VHL; ^†^ significantly different from sprint 4 in the same set, both in RSN and RS-VHL; ^†^ significantly different from sprint 4 in the same set in RSN; ^†^ significantly different from sprint 4 in the same set in RS-VHL; ^‡^ significantly different from sprint 5 in the same set, both in RSN and RS-VHL; ^‡^ significantly different from sprint 5 in the same set in RSN; ^‡^ significantly different from sprint 5 in the same set in RS-VHL; ^#^ significantly different from the sprint with the same number in set 1 both in RSN and RS-VHL

Results are presented in Fig. 4.

The muscle oxygenation variables, O_2_Hb and TOI, decreased with sprinting, while HHb increased (Fig. 4a–c, respectively). The minimum values attained for O_2_Hb or TOI and the maximum values attained for HHb during or just after each sprint were not different between conditions, nor was their pattern of change influenced by the condition. Nevertheless, in each set, the greater drop in O_2_Hb was observed in sprint 2 with a gradual stabilization in the following sprints, and this drop was more pronounced in the 1st set than in the 2nd set, in the first 5 sprints. For HHb, the increase observed augmented from the 1st to the 2nd sprint and then stabilized in both sets. This increase was greater in the 1st set than in the 2nd set. As for O_2_Hb, minimum values of TOI dropped more at sprint 2 and then partially recovered, and the values in the first 4 sprints of the 2nd set are higher than those of the 1st set.

### Rating of perceived exertion and blood lactate concentration

For RPE, there was a significant interaction between sets and conditions (*F*(1, ∞) = 3.991, *p* = 0.046). In RSN, the values increased from set 1 to set 2 (set 1: 7.6 ± 1.3 and set 2: 8.3 ± 1.5, *Z* = 2.586, *p* = 0.010) but not in RS-VHL (set 1: 8.2 ± 1.6 and set 2: 8.6 ± 1.2).

There were no significant differences between conditions in either set. [La] in RS-VHL was lower compared to RSN in both sets (set 1: RS-VHL—7.26 ± 2.65 mmol L^−1^; RSN—7.05 ± 2.20 mmol L^−1^; *p* = 0.020; set 2: RS-VHL—7.78 ± 2.70 mmol L^−1^; RSN—10.34 ± 2.60 mmol L^−1^; *p* = 0.004) and increased from set 1 to set 2 in both conditions (*p* < 0.001). The significant interaction between set and condition (*F*(1.0, 16) = 91.282, *p* = 0.031) suggests that the observed increase from set 1 to set 2 in both conditions was smaller in RS-VHL.

### Neuromuscular fatigue

There was no main effect nor interaction between set and condition for BP_PP_ (RSN: Pre—1533 ± 488.4 W, Post—1428 ± 445.3 W; RS-VHL: Pre—1528 ± 473.9 W, Post—1416 ± 539.1 W).

## Discussion

To the best of our knowledge, this was the first study to compare the effects of an RS protocol with and without the utilization of VHL in an arm cycle ergometer and to evaluate its impact on neuromuscular fatigue.

Performance indicators were significantly lower in RS-VHL when compared to RSN. Decrements in MPO or performance impairments have previously been reported while performing RS in normobaric hypoxia when compared with normoxic conditions (Smith and Billaut 2010; Goods et al. 2014; Morrison et al. 2015). However, the lower values observed in our study in RS-VHL for the performance indicators are not consistent with previous research in VHL (Woorons et al. 2017, 2019a), where the performance indicators were sustained during both RS protocols with and without the application of VHL. Although such an outcome was unexpected, several possible explanations exist for the performance impairments with RSH. These include acute respiratory and metabolic acidosis (Woorons et al. 2007, 2010; Woorons 2014) and the reliance on phosphocreatine resynthesis and H^+^ removal on O_2_-dependent processes (Goods et al. 2014; Morrison et al. 2015). However, we observed lower values of V̇_E_, HR, [La], and RER throughout the RS-VHL protocol, which do not support the first possibility and suggest a greater participation of the aerobic metabolism. The Woorons’ research group suggests that the glycolytic activity is increased with the utilization of the VHL technique, since an increase in [La] was previously observed (Woorons et al. 2011, 2014). It is important to point out that the early VHL protocols have utilized submaximal intensities and shorter rest times (Woorons et al. 2010, 2011; Kume et al. 2016), while the more recent RSH-VHL protocols (Woorons et al. 2017, 2019a), like our protocol, have utilized shorter efforts at higher intensities and longer rest periods. This observation has already been highlighted by Woorons et al. (2021).

While evaluating the oxidative–glycolytic balance during different RS protocols, Raberin et al. (2023) found that in both normoxia and hypoxia, blood lactate concentration increased with the sprint duration, with higher values for 20:40 than for 5:10 effort:pause ratio, suggesting greater dependence on the oxidative component over the glycolytic component in shorter duration sprint protocols such as ours. Considering the importance of the phosphocreatine energy pathway in short-duration RS exercise (Gaitanos et al. 1993), it is reasonable to suggest that the oxidative component may have been preferred throughout the protocol contribution to phosphocreatine resynthesis. Thus, the lower values of [La] and the smaller increase of [La] from set 1 to set 2 in RS-VHL in our study support a smaller utilization of the glycolytic pathway.

In addition, Smith and Billaut (2010) proposed that a decrease in SpO_2_ may contribute to central nervous system fatigue during RSH because of a decline in cerebral oxygenation. This hypothesis arose because of the observation of a decline in both performance and SpO_2_ during RSH with a gradual increase in simulated altitude, which was corroborated by Goods et al. (2014). Conversely, Woorons et al. (2019a), using an RS protocol with VHL, found a decline in cerebral oxygenation that responded faster than the SpO_2_ decline curve; however, the protocol did not negatively affect RSA_decs_. The authors explained the differences between their results and those of Smith and Billaut (2010) given that, unlike in RSH, during the recovery periods following sprints with VHL, SpO_2_ rapidly returns to normal levels (or close), which favors the oxidative process during recovery and consequently the maintenance of performance. In our study, we did not find any SpO_2_ decrease, and so cannot expect a decrement in performance indicators because of significant decline of cerebral oxygenation and consequently central nervous system fatigue. A more plausible explanation could be in line with the findings of Billaut et al. (2023). These authors found that athletes control their pace during all-out sprints, even when they receive strong verbal encouragement, and that this pacing strategy is determined by the task’s endpoint, specifically the number of sprints they have to perform. Although there was a familiarization session, our study participants were not experienced with VHL technique, and even though each session was assigned randomly, the participants were aware of the tasks they were about to perform. In an ideal scenario, a double-blind study would be conducted to eliminate potential biases. However, due to the nature of our study, it was impossible to keep the participants unaware of the task. Therefore, it is possible that this could have, in fact, translated into the performance decrement of MPO, PPO, and TW observed during the RS-VHL protocol and may have largely contributed to the absence of significant differences in SpO_2_.

Previous VHL studies reported that HR drops at the end of each sprint (Woorons et al. 2017, 2019a, b, 2021) but at the end of each rest period, just before each sprint start, during exercise executed at different maximal aerobic velocity percentages until breath-hold breaking point, HR was higher in VHL when compared to RS with unrestricted breathing (Woorons et al. 2021). We cannot carry out such an analysis since we only recorded the higher value during the entire 24-s rest period. According to Woorons et al. (2021), the HR drop at the end of the repetitions with VHL is also a consequence of the large amount of arterial deoxygenation observed in their study which was not seen in our research. Breath-hold training has been widely used, particularly in diving, and a drop in HR has been observed either during exercises with breath-holding with high lung volumes (Lindholm et al. 1999) or during apnoea training without exercise, even in dry conditions (Costalat et al. 2015). This phenomenon can be powerful enough to modify the HR tachycardia induced by exercise (Breskovic et al. 2011) and is considered to be an O_2_-conserving mechanism that results in a decreased cardiac work for reducing the overall ${\dot{\text{V}}}$O_2_ and keeping a sufficient O_2_ supply to the brain (Woorons et al. 2021; Lindholm and Lundgren 2009). Among many other effects, the HR drop results from a primary vagal mechanism within the diving response (Lindholm et al. 1999; Lindholm and Lundgren 2009). In our study, after each sprint bout, whenever VHL was applied, our participants were encouraged to control and slow down their urge to hyperventilate within the time left to rest with deep and slow breaths. Therefore, it is probable that this may have influenced the autonomous nervous system activity (del Negro et al. 2018), which in turn may have influenced the ventilatory response and contributed to the lower V̇_E_ observed in RS-VHL when compared with RSN. Another aspect of the diving response is the enhancement of blood haemoglobin concentration through splenic contraction, which occurs early in the response, even before bradycardia (Lindholm and Lundgren 2009). Exercise interventions and conditions of hypercapnia and hypoxia/hypoxaemia, such as the RS-VHL protocol used in our intervention, can stimulate the spleen to contract (Elia et al. 2021). It was observed that after 3–5 maximal apnoeic attempts, this contraction releases stored erythrocytes into the circulation, enhancing blood’s oxygen-carrying capacity. This increases the oxygen reserve, meaning that subsequent apnoeic bouts start with more available oxygen, slowing oxygen desaturation and extending apnoeic duration (Elia et al. 2021). Moreover, considering that during the pilot study, the 6-s sprint was determined to be the maximal “all-out” duration to maintain VHL breath-hold, it is possible that the participants reached the maximal apnea duration imposed by the exercise intensity and, therefore, experienced splenic contraction. This would explain the lower SpO_2_, the higher ${\dot{\text{V}}}$O_2_ and RER, and the lower [La] values observed in our study in RS-VHL compared to RSN, and again, justify a larger contribution of oxidative processes over the glycolytic processes. It is important to highlight that a recent a study conducted by Ait Ali Braham et al. (2024) using an RS training protocol in combination with VHL, registered only moderate hypoxaemia (92.1%) when compared to RS with unrestricted breathing (97.3%) after each sprint. Given that HR was lower in RS-VHL, the higher increase in ${\dot{\text{V}}}$O_2_ and the stability of HHb from set 1 to set 2 in RS-VHL, when compared with RSN, may be associated with elevated cardiac output, which would be the consequence of higher stroke volume (Woorons et al. 2019a). An increased stroke volume was previously reported during recovery periods following moderate intensity exercise in combination with VHL (Woorons et al. 2011) as a result of a “pump effect” induced by the large and rapid inhalations that occur as soon as the breath-holding ends (Woorons et al. 2019a).

It is only speculative, but it is possible that the combination of a pacing strategy with a less-pronounced diving response by novice users could affect the utilization of VHL by a larger group of participants where individual differences may become more apparent and the potential effects of VHL diluted. This could further lead to misinterpretation of results and underlines the need for a more individualized approach to pacing and strategies of VHL application, namely effort:pause ratios, in future studies.

Due to specificities of the sport, BJJ training may stimulate fast glycolytic fibers to be highly developed in the upper limbs. Furthermore, oxygen extraction and diffusion are critical for muscle function during high-intensity bouts of effort and the arms have shown more significant alterations in blood volume compared to the legs, potentially to better sustain oxygen delivery through the muscle pump and boost perfusion pressure (Willis et al. 2019a, b). Also, the arms showed more significant alterations in deoxyhaemoglobin and total haemoglobin concentrations, along with a higher absolute maximum tissue saturation index than the legs, indicating a superior ability to extract oxygen (Willis et al. 2019a, b) during blow flow restricted exercise. However, the specific differences in oxygen extraction and diffusion between upper and lower limb sprinting during RSH-VHL are not well documented in the literature. We expected to see greater changes in muscle deoxygenation, particularly since the arms had shown to be more sensitive to hypoxia-induced reduction in oxygen supply than the legs (Willis et al. 2019a, b). Woorons et al. (2017) reported greater muscle deoxygenation (higher HHb and lower O_2_Hb) during set 2 of a cycling RSH-VHL protocol when compared to the same exercise in normal breathing conditions, but that was not verified in our study, since HHb was maintained in RS-VHL, while it increased in RSN and it was only possible to see an increase of O_2_Hb from set 1 to set 2, independently of condition. This means that muscle O_2_ extraction and oxygenation were maintained during sprint bouts despite the reduced O_2_ ventilation (Smith and Billaut 2010). The absence of a decrease in SpO_2_ and the greater increase in ${\dot{\text{V}}}$O_2_ in RS-VHL observed in our study may have allowed an adequate influx of oxygen to the muscle, which contributed to the maintenance of HHb in RS-VHL and probably the maintenance of phosphocreatine resynthesis rate, contributing to the absence of differences in the RSA_decs_ between conditions and a smaller decrease in PPO in RS-VHL. Moreover, Raberin et al. (2023) did not find any significant differences in muscle deoxygenation while RS to exhaustion in normoxia or hypoxia, but this was impacted by sprint duration. Therefore, this 6-s short sprint with 24-s rest VHL protocol does not seem to compromise O_2_ availability within the muscle. It should be noted that in the previous studies with VHL and with different degrees of hypoxia, no changes in muscle oxygenation were seen (Smith and Billaut 2010; Billaut et al. 2013; Woorons et al. 2019a). We cannot dismiss the fact that in subsequent studies, researchers have implemented VHL in exercise modalities that engage larger muscle groups, such as running, swimming, and cycling, and therefore, it is possible that the application of VHL to smaller muscle masses, such as our protocol, did not result in the same extent of SpO_2_ reduction, and consequently, it did not significantly influence muscle deoxygenation. This would mean that greater sprint duration could potentially increase the VHL physiological effects. This again highlights the importance of an individualized approach to pacing and effort: pause ratios in future application and research of RS in combination with VHL.

In addition to the previous results, no differences were observed in BP_PP_ after the RS protocol neither in RS-VHL nor in RSN. This may be explained by the nature of the activity, which is a high-intensity explosive movement performed in few milliseconds. Therefore, it recruits high-threshold fast-twitch fibres. Apparently, these were not fatigued nor negatively affected by the RS-VHL protocol. As a movement highly dependent on phosphocreatine availability, again, it is probable that favoring a faster phosphocreatine resynthesis during the RS-VHL protocol and right after it, enough ATP was provided to allow the maintenance of bench press throw peak power after the repeated-sprint protocol.

This can be important for training within combat sports, since the application of these RS protocols, even with VHL, seems not to influence the availability and performance in subsequent high-intensity neuromuscular actions required in a specific training environment. Thus, an athlete can perform an RS session followed by specific combat sports training without performance decrements in high power actions.

There are some limitations of our study. First, the SpO_2_ values were higher than expected and could affect data interpretation. However, we controlled the application of the VHL protocol during data collection and are confident that it was performed correctly. Moreover, we cannot dismiss that a possible pacing effect was present. This underlines that some precautions should be taken into account when applying this technique to novice users, since the familiarization with the VHL technique may have been too short and may have influenced the experimental results. Furthermore, the group was not homogeneous regarding competitive level or training experience, specifically regarding strength training. Additionally, the NIRS probe placement in the long head of the triceps brachii may not represent the measured variable in all the muscles involved in the arm ergometer action.

## Conclusions

The main finding of this study is that a repeated-sprint exercise performed with VHL in an arm-crank ergometer did not induce a significant drop in SpO_2_ and, consequently, did not induce a drop in muscle oxygenation. Performance indicators, PPO, MPO, and TW, were significantly affected by RS-VHL, suggesting that this must be taken into account by coaches and sports physiologists if they decide to use RS-VHL over traditional RS. Conversely, RSA_decs_ was not affected. This is the first study to introduce a fatigue evaluation of explosive movements to analyse the impact of the RS protocol in both conditions, and no effect was observed. These two parameters combined suggest that the VHL technique did not affect the neuromuscular performance after the application of the technique, indicating that it is possible, apart from concurrent considerations regarding the specificity of the stimuli, to include this protocol in a common combat sports training session.

[La] and RER were lower in RS-VHL when compared with RSN. These results combined suggest that the aerobic system may have been preferentially utilised over the glycolytic pathway.

Our subjects were found to use a pacing strategy and this could have affected the outcomes. Furthermore this highlights the need for a more individualized approach to pacing and strategies of VHL application, in particular effort:pause ratios, in future training and VHL research.

## Data Availability

Data will be made available upon reasonable request.

## Abbreviations

BJJ: Brazilian Jiu-Jitsu
BP_PP_: Bench press throw peak power
HHb: Deoxygenated haemoglobin/myoglobin
HG_Iso_: Hand grip isometric strength
HR: Heart rate
[La]: Blood lactate concentration
MPO: Mean power output
NIRS: Near Infrared Spectroscopy
O_2_Hb: Oxygenated haemoglobin/myoglobin
PPO: Peak power output
RER: Respiratory exchange ratio
RPE: Rating of perceived exertion
RS: Repeated sprints
RSA_decs_: Repeated sprints percentage decrement score
RSH: Repeated sprints in hypoxia
RS-VHL: Repeated sprints with voluntary hypoventilation at a low lung volume
RSN: Repeated sprints with normal breathing
SpO_2_: Arterial oxygen saturation
TOI: Tissue oxygenation index
TW: Total work
${\dot{\text{V}}}$CO_2_: Volume of carbon dioxide exhaled
$${\dot{\text{V}}}$$_E_: Ventilation
${\dot{\text{V}}}$CO_2_: Oxygen uptake
${\dot{\text{V}}}$O_2peak_: Peak oxygen uptake
${\dot{\text{V}}}$O_2__r__el_: Volume of oxygen inhaled per kg of body weight
VHL: Voluntary hypoventilation at low lung volume
